# Anti-cancer effect and potential microRNAs targets of ginsenosides against breast cancer

**DOI:** 10.3389/fphar.2022.1033017

**Published:** 2022-10-05

**Authors:** Meiling Fan, Mengyao Shan, Xintian Lan, Xiaoxue Fang, Dimeng Song, Haoming Luo, Donglu Wu

**Affiliations:** ^1^ Changchun University of Chinese Medicine, Changchun, China; ^2^ School of Pharmacy, Changchun University of Chinese Medicine, Changchun, China; ^3^ Key Laboratory of Effective Components of Traditional Chinese Medicine, Changchun, China; ^4^ School of Clinical Medical, Changchun University of Chinese Medicine, Changchun, China

**Keywords:** ginsenosides, breast cancer, MicroRNAs, combination therapy, molecular mechanism

## Abstract

Breast cancer (BC) is one of the most common malignant tumor, the incidence of which has increased worldwide in recent years. Ginsenosides are the main active components of *Panax ginseng* C. A. Mey., *in vitro* and *in vivo* studies have confirmed that ginsenosides have significant anti-cancer activity, including BC. It is reported that ginsenosides can induce BC cells apoptosis, inhibit BC cells proliferation, migration, invasion, as well as autophagy and angiogenesis, thereby suppress the procession of BC. In this review, the therapeutic effects and the molecular mechanisms of ginsenosides on BC will be summarized. And the combination strategy of ginsenosides with other drugs on BC will also be discussed. In addition, epigenetic changes, especially microRNAs (miRNAs) targeted by ginsenosides in the treatment of BC are clarified.

## Introduction

Ginsenosides are the main active components of traditional Chinese herbal medicine *Panax ginseng* C. A. Mey. (Song et al., 2022). At present, nearly 200 ginsenosides have been isolated and identified from the roots, stems, leaves, flowers buds, and berries of *Panax ginseng* C. A. Mey. (Zhao A. et al., 2022). Ginsenosides can be divided into three classes according to different aglycone structures, protopanaxadiol (PPD), protopanaxatriol (PPT) and oleanolic acid. PPD types mainly includes ginsenosides Rb1, Rd, Rg3, Rh2, CK and F2, etc., PPT types mainly includes ginsenosides Re, Rf, Rg1, Rg2, Rh1 and F1, etc., and oleanolic acid types mainly includes ginsenosides Ro, etc. (Hou et al., 2021; Liu G. et al., 2022). Modern pharmacology has shown that ginsenosides have neuroprotective (Zarneshan et al., 2022), anti-aging (Meng et al., 2022), anti-oxidant (He et al., 2022), anti-inflammatory (Xu et al., 2022) and anti-cancer (Zhao L. et al., 2022) effects. Numerous ginsenosides has been reported have various anti-cancer activity. Such as, ginsenoside Rg3 can effectively inhibit prostate cancer, gastric cancer, gallblader cancer and ovarian cancer, etc., while ginsenoside CK can function in suppress the proliferation and procession of liver cancer, lung cancer, colon cancer, and bladder cancer, etc. (Liu Z. et al., 2021; Liu J. et al., 2022).

Breast cancer (BC) is the most common cancer among female patients in the world (Zeng et al., 2020). According to the data of International Agency for Research on Cancer (IARC) (Hu et al., 2022), more than 2.26 million women in the world were diagnosed with BC and nearly 68.5 million women died of BC in 2020. While this number was estimated around more than 3 million new cases and 1 million deaths every year by 2040. BC is a disease with complex etiology and high heterogeneity, can be divided into three types, hormone receptor positive (estrogen and progesterone), human epidermal growth factor receptor 2 (HER2) positive and triple negative breast cancer (TNBC, accounting for 10%–20% of BC cases) (Karami Fath et al., 2022). In clinical, treatment of BC mainly include surgical resection, radiotherapy, hormone therapy and so on (Qiu et al., 2021). However, these treatments are accompanied by adverse conditions such as drug resistance (Hobbs et al., 2022).

Studies have shown that ginsenosides can mediating numerous processions of BC, including drive apoptosis and autophagy, regulate cell cycle and inhibit metastasis (Jin et al., 2020). At present, a variety of ginsenosides have been reported to inhibit the proliferation of BC, such as ginsenoside Rg3 and ginsenoside Rd can inhibit the metastasis of BC cells. While ginsenosides Rg2, Rg5 and CK can induce autophagy, apoptosis and cell cycle arrest of BC cells (Hong et al., 2021). Moreover, combination treatment of ginsenosides and other chemotherapeutic drugs have been reported. For example, combination of ginsenoside Rg3 with curcumin or endostar can improve BC cells radiosensitivity, and inhibit metastasis of BC cells, respectively (Zhang et al., 2016; Changizi et al., 2021). The relationship between microRNAs (miRNAs) and BC was first clarified in 2005, and subsequent studies revealed numerous miRNAs closely related to the development of BC (Iorio et al., 2005; Shekari et al., 2022). It has been reported that miRNAs can be used to determine the stage of BC, thereby predicting the survival rate of BC patients (Karami Fath et al., 2022). Moreover, miRNAs detected in body fluids serve as biomarkers for the diagnosis and prognosis of BC (Liu L. et al., 2022). Ginsenoside Rd inhibited the expression of miR-18a, which in turn inhibited the proliferation, metastasis and invasion of BC cells (Wang et al., 2016). While ginsenoside Rh2 inhibits proliferation and induces apoptosis of BC cells by inhibiting miR-4425 and miR-3614-3p expression, respectively (Park et al., 2021; Park et al., 2022). The present review is aim to summarize the pharmacological activities of ginsenosides in BC therapy, as well as indicated molecular mechanism. Further overview the combination therapies against BC and discuss the clinical prospects of ginsenosides. Moreover, miRNAs targeted by ginsenosides in BC treatment will also be demonstrated.

## Anti-breast cancer activity of ginsenosides

**FIGURE 1:**
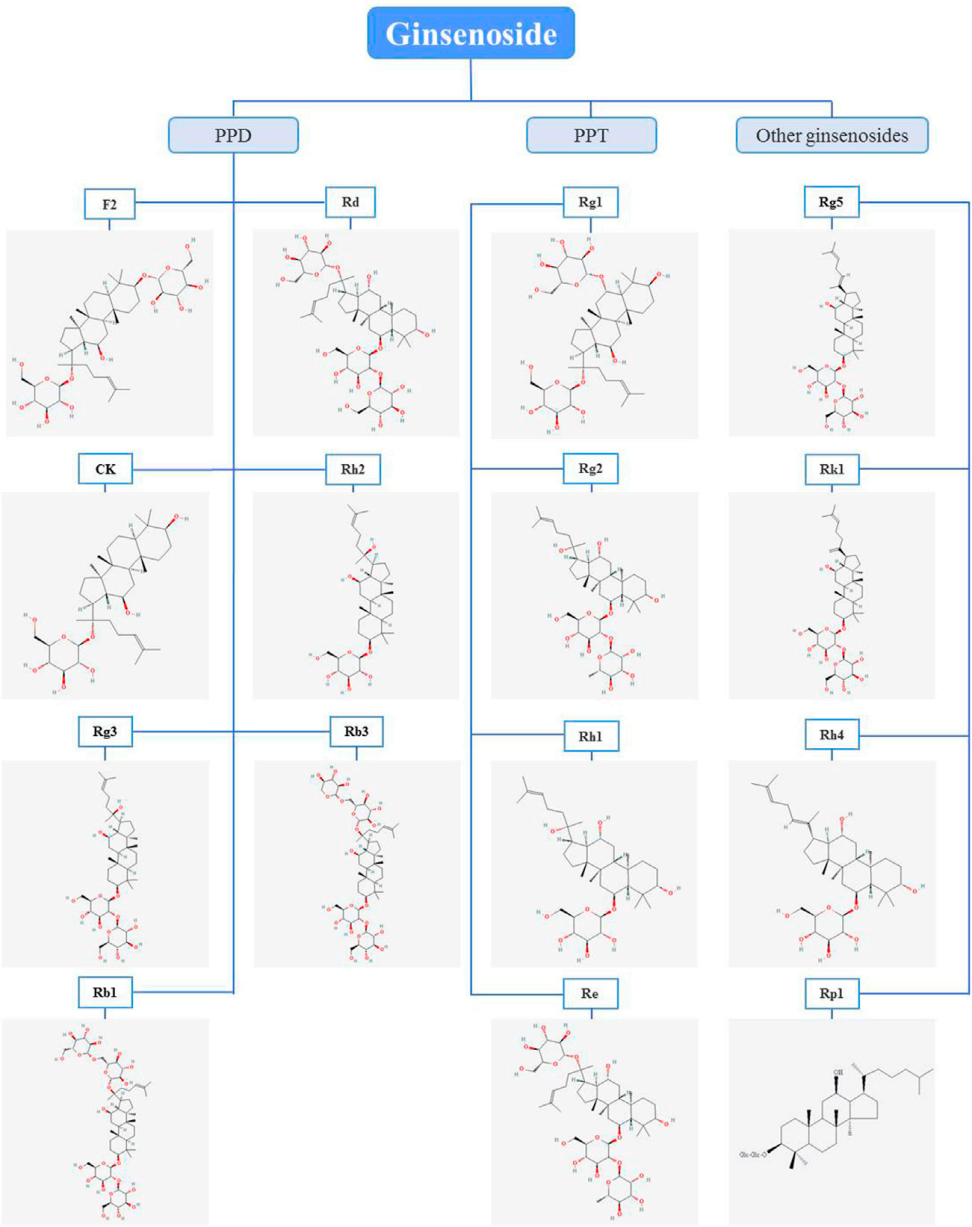
Chemical structures of ginsenosides with anti-breast cancer activity. Glc, glucose. The chemical structures of the ginsenosides included in this publication were obtained from pubchem, but ginsenoside Rp1 was drawn using the chemdraw program.

**FIGURE 2:**
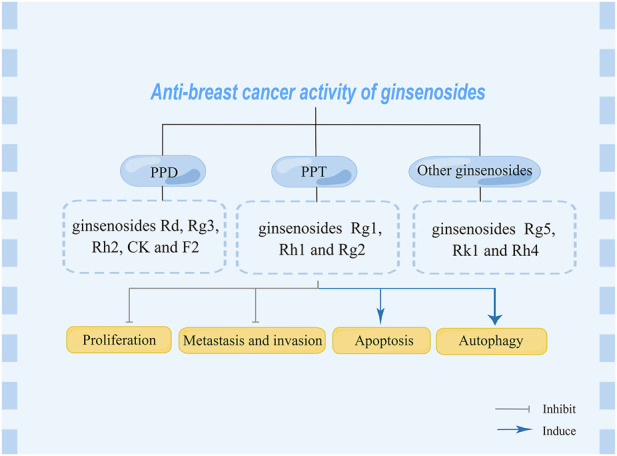
Anti-breast cancer effect of various ginsenosides.

PPD and PPT are both dammarane tetracyclic triterpene saponins, and the main difference in structure is whether there is a hydroxyl substitution on the carbon at position 6 (Pan et al., 2018). Moreover, in the anti-BC research of ginsenosides, it mainly revolves around PPD and PPT types ginsenosides, and in details, the structure is mainly different in groups on the carbon at positions 3, 6 and 20 (Figure 1). In the following section, the biological activity of ginsenosides against BC according to ginsenosides chemical structures will be investigated (Figure 2, Table 1).

### Anti-breast cancer activity of PPD types

PPD has good biological activities of anti-oxidation, anti-inflammatory, as well as anti-cancer (Yang et al., 2019). At present, several studies have reported the anti-BC effects of PPD and related mechanisms. Such as, ginsenoside F2 inhibits the proliferation of breast cancer stem cells (CSCs) by regulating p53 to induce apoptosis and stimulate autophagy (Mai et al., 2012). Ginsenoside Rb1 can decrease tumor growth and tumor weight *in vivo*, and induce apoptosis of BC cells *in vitro* by binding to carbon nanotubes (Lahiani et al., 2017; Zuo et al., 2022). Moreover, ginsenoside Rb3 increases the anti-proliferative activity of cisplatin at higher concentrations (Aung et al., 2007). In this report, the berry extract of American ginseng has the effect of anti-proliferation of BC cells, and ginsenoside Rb3 is the active ingredient with the highest content. We speculate that ginsenoside Rb3 has the effect of anti-BC.

***Ginsenoside Rd*** Ginsenoside Rd is one of the metabolites of ginsenoside Rb1 in the intestine (Zhang et al., 2021) and has a wide range of biological activities including neuroprotection, improved metabolism, as well as anti-cancer (Li J. et al., 2022). It is reported that ginsenoside Rd inhibits proliferation and drives apoptosis of BC cells (Kim 2013). Ginsenoside Rd can target transient receptor potential (TRP) melastatin 7 (TRPM7), a member of the TRP channel family and functions in cell cycle regulation, further mediate the proliferation and survival of BC cells (Liu H. et al., 2020). On the other hand, ginsenoside Rd shows the activity of suppressing angiogenesis and metastasis. In details, ginsenoside Rd inhibited migration, invasion and lung metastasis of BC cells *in vitro* and *in vivo*, respectively (Wang et al., 2016). Moreover, ginsenoside Rd can inhibit HIF-1α (hypoxia inducible factor-1)/VEGF (vascular endothelial growth factors) through Akt/mTOR/p70S6K signaling pathway and then inhibit angiogenesis and cut off nutrient supply (Zhang et al., 2017). VEGF, also known as vascular permeability factor, promotes endothelial cell proliferation, migration and invasion into surrounding tissues, and is an important factor in tumor angiogenesis (Adams and Alitalo 2007). Therefore, we speculate that ginsenoside Rd can not only inhibit angiogenesis but also inhibit the growth, metastasis and invasion of BC cells by targeting VEGF.

***Ginsenoside CK*** Ginsenoside CK is the main metabolite and final absorption form of PPD type ginsenoside in the intestine (Jin et al., 2018). Ginsenoside CK can inhibit the proliferation, induce apoptosis of MCF-7 cells, as well as EMT, through down-regulating the PI3K/Akt pathway (Zhang and Li 2016). Moreover, ginsenoside CK can reduce glycogen synthase kinase-3β (GSK-3β) phosphorylation, which leads to decreased expression of oncoprotein *β*-catenin and cyclin D1, thereby induce programmed necrosis of cancer cells (Kwak et al., 2015). TNBC is particularly addicted to glutamine, which is an essential nutrient that replenishes energy to cancer cells (Edwards et al., 2021). Studies have shown that ginsenoside CK targets glutamine metabolism and exerts anti-cancer effects on high glutamine-dependent TNBC cells both *in vitro* and *in vivo*. In particularly, ginsenoside CK decreased the expression of glutaminase 1 (GLS1), resulting in reduced ATP production, attenuated amino acid availability, causing oxidative stress and TNBC cells growth inhibition and apoptosis (Zhang B. et al., 2022).

***Ginsenoside Rh2*** Ginsenoside Rh2 has several pharmacological activities, including improving cardiac function, anti-inflammatory and anti-cancer effects (Liu T. et al., 2022). It is reported that ginsenoside Rh2 mediates cell cycle arrest and inhibits proliferation of BC cells (Oh et al., 1999; Hu et al., 2021). Ginsenoside Rh2 can mediate G_1_/S cell cycle arrest by activating p38 and decreasing cyclin D1 expression (Peng et al., 2022). Moreover, ginsenoside Rh2 mediates G1 phase arrest of BC cells, which is caused by p15 INK4B and p27 kip1 dependent inhibition of CDKs/cyclin complex (Choi et al., 2009). In addition, ginsenoside Rh2 can up-regulate the phosphorylation levels of p53, p38 and ASK1 (apoptosis signal-regulating kinase 1), whereas down-regulate the expression of TRAF2 (TNF receptor-associated factor 2), thereby inhibiting the proliferation of BC cells (Ren et al., 2018). It is reported that ginsenoside Rh2 can induce BC cells apoptosis (Park et al., 2022). Studies have shown that ginsenoside Rh2 can increase the expression of TNFα (tumor necrosis factor α) by up-regulating ERβ (estrogen receptor *β*), which in turn induces apoptosis of BC cells (Peng et al., 2022). Furthermore, ginsenoside Rh2 induced apoptosis of BC cells was associated with increased levels of pro-apoptotic proteins including Bak, Bax and Bim, and decreased levels of anti-apoptotic proteins including Bcl-2, Bcl-xL and Mcl-1 (Choi et al., 2011). On the other hand, ginsenoside Rh2 shows the activity of inhibiting the migration and invasion of cancer cells. In details, studies have shown that ginsenoside Rh2 can inhibit migration and invasion of BC cells promoted by the senescence-associated secretory phenotype of BC cells and normal epithelial cells (Hou et al., 2019).

***Ginsenoside Rg3*** Ginsenoside Rg3 has anti-cancer activity and has been demonstrated in many types of cancer, including BC (Liu M. Y. et al., 2021). It has been reported that two epimers of ginsenoside Rg3, 20(S)-ginsenoside Rg3 (SRg3) and 20(R)-ginsenoside Rg3 (RRg3), can inhibit the proliferation, migration and invasion of BC cells, respectively (Nakhjavani et al., 2019). In details, ginsenoside Rg3 can affect cell proliferation through a variety of pathways, including inducing protein synthesis, cell division, and inhibiting nuclear factor kappa-B (NF-κB) signaling (Zou et al., 2018), among which, SRg3 inhibits BC cells proliferation by arresting the cell cycle of G0/G1 (Nakhjavani et al., 2019). Furthermore, studies demonstrated that SRg3 induces apoptosis of BC cells through classical mitochondria-dependent caspase activation (Kim et al., 2013). In details, SRg3 inhibits the phosphorylation of ERK and Akt, as well as induces the instability of mutant p53, thereby blocking NF-κB signal transduction, which in turn decreases the expression of Bcl-2 and ultimately induces apoptosis of BC cells (Kim et al., 2014). Moreover, ginsenoside Rg3 shows the activity of inhibiting angiogenesis, driving autophagy, as well as mitastasis of BC cells (Zhang et al., 2016). SRg3 inhibits the expression of VEGFs and matrix metalloproteinases (MMPs), which in turn suppresses angiogenesis, and further enhances the autophagic process by inhibiting mTOR/PI3K/Akt and JNK/Beclin-1 signaling pathways (Zhang et al., 2016). While SRg3 can also target CXCR4 expression and function in inhibiting the migration of BC cells (Chen et al., 2011). On the other hand, ginsenoside Rg3 are able to reduce stem cell properties and EMT to exert anti-cancer effects. Cancer stem cell properties play critical roles in tumorigenesis, progression, and therapy, and SRg3 inhibits self-renewal activity in breast stem-like cancer cells by blocking Akt-induced HIF-1α activation and inhibiting HIF-1α-regulated expression of Bmi-1 and Sox-2 (Oh et al., 2019). Moreover, SRg3 inhibited PI3K/AKT pathway to decrease the expression of cellular “stemness”, which in turn reduced mammosphere formation efficiency (Nakhjavani et al., 2021). And studies have shown that (S, R) Rg3 down-regulates cancer “stemness” and EMT suppresses myeloid-derived suppressor cell (MDSC) of BC cells, thereby exerting anti-cancer effects (Song et al., 2020).

### Anti-breast cancer activity of PPT types

PPT has anti-cancer biological activity, including BC (Yu et al., 2018; Wang B. et al., 2020). For example, ginsenoside Re has the activity of inhibiting the proliferation of BC cells, while increasing the activity of cisplatin in a concentration-related manner and improving its anti-proliferative effect on BC (Aung et al., 2007).

***Ginsenoside Rg1*** Ginsenoside Rg1 is an effective anti-cancer component in total ginsenosides (Li J. et al., 2014). It is reported that ginsenoside Rg1 decreases cell viability, inhibits cell proliferation, induces reactive oxygen species (ROS) thereby drives apoptosis of BC cells (Chu et al., 2020). In details, ginsenoside Rg1 induces apoptosis by generating ROS of BC cells (Chu et al., 2020). On the other hand, ginsenoside Rg1 shows the activity of suppressing invasion and migration of BC cells. Studies have shown that ginsenoside Rg1 inhibits PMA-induced MMP-9 expression by inhibiting NF-κB activity, which in turn inhibits invasion and migration of BC cells (Li L. et al., 2014). Moreover, ginsenoside Rg1 can down-regulate the expression of angiogenesis and EMT markers, thereby functions in inhibiting angiogenesis and EMT (Chu et al., 2020). Therefore, we speculate that ginsenoside Rg1 has the effect of blocking BC cells metastasis mediated by EMT.

***Ginsenoside Rg2*** Ginsenoside Rg2 has the functions of enhancing memory, improving metabolism, protecting heart, as well as anti-cancer (Liu X et al., 2022). It is reported that ginsenoside Rg2 induces cell cycle arrest, inhibits proliferation and drives apoptosis of BC cells (Jeon et al., 2021a). In details, ginsenoside Rg2 can induce ROS production by inhibiting the activation of ERK1/2 and Akt, which in turn inhibits proliferation of BC cells, further arrest the cell cycle of G1 and induce apoptosis of BC cells by promoting ROS-mediated AMPK activation (Jeon et al., 2021b). In addition, studies have shown that ginsenoside Rg2 can mediate the activation of p53, which in turn induces the up-regulation of AMPK, further functions in regulating apoptosis and autophagy of BC cells (Chung et al., 2018).

***Ginsenoside Rh1*** Ginsenoside Rh1 is a metabolite of ginsenoside Re and Rg1, and has been reported to possess anti-allergic, anti-inflammatory, anti-aging, anti-oxidant and anti-cancer activity (Lyu et al., 2019). It is reported that ginsenoside Rh1 induces cell cycle arrest and drives apoptosis of BC cells (Huynh et al., 2021; Jin et al., 2022). In details, ginsenoside Rh1 induces cell cycle arrest, apoptosis, as well as autophagy by inhibiting ROS mediated PI3K/Akt pathway (Huynh et al., 2021). Moreover, ginsenoside Rh1 increases cell cycle arrest and apoptosis by inducing mtROS (mitochondrial ROS)-activated intracellular calcium accumulation and ER stress signaling pathways (Jin et al., 2022). On the other hand, studies have shown that ginsenoside Rh1 increases the production of mtROS to induce mitochondrial dysfunction, thereby inhibiting STAT/NF-κB signaling pathway, which in turn inhibits migration, invasion of BC cells, as well as angiogenesis (Jin et al., 2021).

### Anti-breast cancer activity of other ginsenosides

At present, rare ginsenosides such as Rg5, Rk1, Rh4 have also begun to be extensively studied, which are the deglycosylated forms of the main ginsenosides, and harvest greater drug potential (Qi et al., 2011). Ginsenoside Rg5 is a minor ginsenoside synthesized by deglycylation of ginsenoside Rb1 and dehydration of the carbon at the 20-position of ginsenoside Rg3 during ginsenoside cooking treatment, and exhibits excellent anti-BC effect (Liu M. Y. et al., 2021). Numerous studies have shown that ginsenoside Rg5 can induce apoptotic death in BC cells, one by inhibiting the PI3K/Akt/mTOR pathway and subsequently reducing Bad phosphorylation (Liu and Fan 2018; Liu and Fan 2020), and the other by regulating mitochondria-mediated pathway to induce caspase-dependent apoptosis including caspase-3, caspase-8, caspase-9 and Poly (ADP-ribose) polymerase (PARP) (Kim and Kim 2015; Liu and Fan 2018; Liu and Fan 2020). Moreover, ginsenoside Rg5 can up-regulate the expression of LC3-II, Beclin-1, Atg5 and Atg12 and down-regulate the expression of p62, thereby inducing autophagy and promoting BC cells death (Liu and Fan 2018; Liu and Fan 2020). Ginsenoside Rg5 can also induce G0/G1 cell cycle arrest by reducing the protein expression of cyclin D1, cyclin E2 and CDK4, and increasing the expression of p15 ink 4B, p53 and p21WAF1/CIP1, and subsequently inhibit the proliferation of BC cells (Kim and Kim 2015). Ginsenoside Rk1 is derived from ginsenoside Rg3 through a dehydration or heating process (Ryoo et al., 2020). The study found that ginsenoside Rk1 can also inhibit the growth of TNBC cells by inhibiting the expression of cycle-related proteins, causing cells to arrest in G0/G1 phase (Hong and Fan 2019). In addition, ginsenoside Rk1 can also regulate the production of intracellular ROS and reduce mitochondrial membrane potential, and then increase the expression of Bax and cytochrome C, thereby inducing apoptosis and death of TNBC cells (Hong and Fan 2019). Ginsenoside Rh4 is produced by Rg1 and Re by restoring antioxidant defense enzyme activity or inhibiting ROS generation (Baek et al., 2017), and has been shown to have inhibitory effects on the occurrence of colorectal cancer, lung cancer and esophageal cancer (Baek et al., 1996). Daidi Fan’s research shows that ginsenoside Rh4 can not only effectively inhibit the proliferation of S-phase cells, but also induce apoptosis by reducing Bcl-2, increasing Bax and activating caspase-8, caspase-3 and PARP, thereby inhibiting BC cells growth (Duan et al., 2018). On the other hand, ginsenoside Rp1 is prepared from ginsenosides Rg5, Rk1, etc. through reduction and hydrogenation reactions (Cho et al., 2001). It has been reported that ginsenoside Rp1 can induce cell cycle arrest and apoptosis of BC cells through inhibit the insulin-like growth factor 1 receptor (IGF-1R)/Akt pathway (Kang et al., 2011). In details, MCF-7 cells were arrested in G1 phase and MDA-MB-231 and T-47D cells were arrested in G2/M phase. In this study, Ju-Hee Kang et al. proposed that ginsenoside Rp1 could be further investigated to inhibit metastasis of BC cells.

## Anti-breast cancer activity of ginsenosides mediated by microRNAs

MiRNAs are a group of endogenous noncoding RNAs that regulate gene expression, ranging in size from 19 to 25 nucleotides (Lu and Rothenberg 2018). MiRNAs bind to the 3′-UTR (untranslated region) of a specific target gene to degrade mRNA or inhibit its protein translation (Lee and Dutta 2009). In general, miRNAs are aberrantly expressed in a variety of cancers, oncogenic miRNAs are frequently overexpressed, and tumor suppressor miRNAs are frequently downregulated (Fridrichova and Zmetakova 2019). A growing number of studies have shown that some miRNAs are emerging as BC diagnostic, prognostic and therapeutic biomarkers and control BC hallmark functions, such as invasion, metastasis, proliferation and apoptosis, etc (Bertoli et al., 2015; Xu et al., 2020). For example, the miR-200 family is often present in BC cells as tumor suppressors, regulating EMT by targeting the transcriptional repressors ZEB1 and ZEB2, thereby inhibit the metastasis and invasion of BC cells (Liu 2012).

It has also been reported that ginsenosides can inhibit the occurrence and growth of cancer cells by targeting miRNAs (Table 2). Studies have shown that ginsenoside Rh2 can inhibit the proliferation of lung cancer cells and glioma cells by targeting miR-491 and miR-128 (Wu et al., 2011; Chen et al., 2019). Ginsenoside Rg3 regulates the expression of target genes by targeting miRNAs including miR-4425, miR-603 and miR-324-5p, which in turn inhibits ovarian cancer progression (Zheng et al., 2018; Lu et al., 2019; Lu et al., 2020). Ginsenosides Rh7 and Rg1 have been reported to inhibit the progression of non-small cell lung cancer (NSCLC) by targeting miR-212 and miR-126, respectively (Chen et al., 2021a; Chen et al., 2022). In particularly, several studies clarify that ginsenosides mediate the miRNAs expression profile of BC (Li et al., 2020). For example, ginsenoside Rh2 can up-regulate LncRNA STXBP5-AS1, whereas inhibit the level of miR-4425, further reduce the expression of target gene RNF217, thereby inhibit the growth of BC cells (Park et al., 2021). Ginsenoside Rh2 can also down-regulate the expression of miR-3614-3p mediated by CFAP20DC-AS1, and then reduce the expression of BBX and TNFAIP3, thereby inducing apoptosis of BC cells (Park et al., 2022). Moreover, ginsenoside Rh2 can reduce the drug resistance of BC by targeting miR-222, miR-34a and miR-29a to down-regulate the expression of target gene Bax (Wen et al., 2015). According to reports, ginsenoside Rd can down-regulate the expression of Smad2 by targeting miR-18a, thereby inhibit the metastasis and invasion of BC cells (Wang et al., 2016). Ginsenoside Rg3 can activate oncogenic CHRM3 and DACH1 by downregulating ATXN8OS-mediated miR-424-5p, thereby inhibiting BC cells proliferation (Kim et al., 2021). It has been reported that ginsenosides Rg3 can block the occurrence of EMT in ovarian cancer cells by targeting miR-145, which has been shown to play an important role in inhibiting the migration and invasion of BC cells by directly targeting the angiopoietin 2 gene (ANGPT2) of BC cells (Li et al., 2017; Liu et al., 2017; Jiang et al., 2019; Tang et al., 2019). Therefore, we speculate that ginsenoside Rg3 inhibits the growth of BC cells by regulating miR-145, which needs further demonstrated.

**TABLE 1:** Inhibition of ginsenosides on breast cancer cells through inducing cell cycle arrest and apoptosis, inhibiting proliferation, migration, invasion, autophagy, angiogenesis and EMT, etc.

Type of ginsenoside	Physiological effects	Cell lines	Related signaling pathways	References
Rh2	Arrest cell cycle	MCF-7, MDA-MB-231	N/A	(Choi et al., 2009; Peng et al., 2022)
Rg2		MCF-7	N/A	(Jeon et al., 2021a; Jeon et al., 2021b)
Rh1		MCF-7, HCC1428; MDA-MB-231	Inhibit ROS/PI3K/Akt pathway; Induce intracellular calcium accumulation and ER stress signaling pathways	(Huynh et al., 2021; Jin et al., 2022)
Rg5		MCF-7	N/A	Kim and Kim (2015)
Rk1		MDA-MB-231	N/A	Hong and Fan (2019)
Rp1		MCF-7, T-47D, MDA-MB-231	Inhibit the IGF-1R/Akt pathway	Kang et al. (2011)
F2	Inhibit proliferation	CSCs	N/A	Mai et al. (2012)
Rd		MCF-7; MDA-MB-231	Inhibit TRPM7	(Kim 2013; Liu H. et al., 2020)
CK		MCF-7	Inhibit PI3K/Akt pathway	Zhang and Li (2016)
**Type of ginsenoside**	**Physiological effects**	**Cell lines**	**Related signaling pathways**	**References**
Rh2		MCF-7, MDA-MB-231	N/A	Ren et al. (2018)
Rg3	Induce apoptosis	MDA-MB-231	N/A	Nakhjavani et al. (2019)
Rg1		MDA-MB-231	N/A	Chu et al. (2020)
Rg2		MCF-7	N/A	(Jeon et al., 2021a; Jeon et al., 2021b)
Rh4		MCF-7	N/A	Duan et al. (2018)
Rd		MCF-7	N/A	Kim (2013)
Rp1		MCF-7, T-47D, MDA-MB-231	Inhibit the IGF-1R/Akt pathway	Kang et al. (2011)
CK	Inhibit proliferation	MCF-7; TNBC cells (MDA-MB-231, etc.)	Inhibit PI3K/Akt pathway	(Zhang and Li 2016; Zhang B. et al., 2022)
Rh2		MCF-7, MDA-MB-231	N/A	(Choi et al., 2011; Peng et al., 2022)
Rg3		MDA-MB-231	Block NF-κB signal transduction	(Kim et al., 2013; Kim et al., 2014)
**Type of ginsenoside**	**Physiological effects**	**Cell lines**	**Related signaling pathways**	**References**
Rg1		MDA-MB-231	N/A	Chu et al. (2020)
Rg2		MCF-7	N/A	(Jeon et al., 2021a; Jeon et al., 2021b)
Rh1		MCF-7, HCC1428; MDA-MB-231	Inhibit ROS/PI3K/Akt pathway; Induce intracellular calcium accumulation and ER stress signaling pathways	(Huynh et al., 2021; Jin et al., 2022)
Rg5		MCF-7	Inhibit the PI3K/Akt/mTOR pathway; Regulat mitochondria-mediated pathway	(Kim and Kim 2015; Liu and Fan 2018; Liu and Fan 2020)
Rk1		MDA-MB-231	Inhibit ROS/PI3K/Akt pathway	Hong and Fan (2019)
Rh4		MCF-7	N/A	Duan et al. (2018)
Rd	Inhibit migration and invasion	4T1 (*in vitro* and *in vivo*)	N/A	Wang et al. (2016)
Rh2		MDA-MB-231	N/A	Hou et al. (2019)
Rg3		MDA-MB-231	N/A	Chen et al. (2011)
**Type of ginsenoside**	**Physiological effects**	**Cell lines**	**Related signaling pathways**	**References**
Rg1		MCF-7	Inhibit NF-κB activity	Li J. et al. (2014)
Rh1	Induce autophagy	MDA-MB-231	Inhibit STAT/NF-κB signaling pathway	Jin et al. (2021)
Rg3		MCF-7	Inhibit mTOR/PI3K/Akt and JNK/Beclin-1 signaling pathways	Zhang et al. (2016)
Rg2		MCF-7	N/A	Chung et al. (2018)
Rh1		MCF-7, HCC1428	Inhibit ROS/PI3K/Akt pathway	Huynh et al. (2021)
Rg5		MCF-7	N/A	(Liu and Fan 2018; Liu and Fan 2020)
Rd	Inhibit angiogenesis Inhibit EMT	MDA-MB-231	Inhibit Akt/Mtor/p70S6K pathway	Zhang et al. (2017)
Rg3		MCF-7	N/A	Zhang et al. (2016)
CK		MCF-7	Inhibit PI3K/Akt pathway	Zhang and Li (2016)
Rg3		MDSCs	N/A	Song et al. (2020)

**TABLE 2:** Ginsenoside regulation of miRNAs in breast cancer.

Type of ginsenoside	Type of cancer	miRNAs	miRNA targets	References
Rh2	BC	miR-4425↓	RNF217↓	Park et al. (2021)
	BC	miR-222↓	Bax↓	Wen et al. (2015)
	BC	miR-34a↓	Bax↓	Wen et al. (2015)
	BC	miR-29a↓	Bax↓	Wen et al. (2015)
	BC	miR-3614-3p↓	BBX and TNFAIP3↓	Park et al. (2022)
Rd	BC	miR-18a↓	Smad2↓	Wang et al. (2016)

It has been reported that ginsenosides Rg3 can block the occurrence of EMT in ovarian cancer cells by targeting miR-145, which has been shown to play an important role in inhibiting the migration and invasion of BC cells by directly targeting the angiopoietin 2 gene (ANGPT2) of BC cells (Li et al., 2017; Liu et al., 2017; Jiang et al., 2019; Tang et al., 2019). Therefore, we speculate that ginsenoside Rg3 inhibits the growth of BC cells by regulating miR-145, which needs further demonstrated. Moreover, ginsenoside Rg3 can improve gastric precancerous lesions by targeting miR-21 (Liu W. et al., 2020), which has been shown to regulate the proliferation and invasion of BC (Ali et al., 2020) and TNBC cells (Fang et al., 2017). Afterwards, we speculate that ginsenoside Rg3 targeting miR-21 to regulate the growth of BC and TNBC requires more clinical studies. Notably, targeting miR-126 expression has been shown to inhibit PI3K/AKT signaling activity and thereby inhibit BC cells growth (McGuire et al., 2015). Ginsenoside Rg1 can also inhibit NSCLC by targeting miR-126 to inhibit the PI3K/AKT pathway (Chen et al., 2022). Therefore, we speculate that ginsenoside Rg1 may regulate the PI3K/AKT pathway by targeting miR-126, thereby inhibiting the progression of BC, which needs more experiments to confirm. Numerous studies have shown that ginsenoside Rh2 can effectively inhibit IL-6-induced STAT3 phosphorylation and the expression of miR-214 in cultured normal colonic epithelial cells to relieve ulcerative colitis (Chen et al., 2021b). The regulation of miRNA-214 can also inhibit the biological activity of breast cancer cells (Ouyang et al., 2020). It can be speculated that ginsenoside Rh2 may inhibit the growth of BC by targeting miRNA-214.

In conclusion, ginsenosides have great potential as broad-spectrum anti-cancer drugs and effective chemosensitizers.

## Future prospects

Currently, surgery, radiotherapy and chemotherapy are the main methods used for clinical cancer treatment (Colli et al., 2017). However, serious toxic and side effects of chemoradiotherapy drugs and multidrug resistance (MDR) interfered with therapeutic effect. (Colli et al., 2017). Conversely, combination therapy can reduce the incidence of drug resistance in cancer cells by targeting different pathways, and can reduce toxicity by reducing the required dose of a single drug (Aumeeruddy and Mahomoodally 2019). It is reported that ginsenosides can inhibit the growth of cancer cells through different pathways and can be used as adjuvant drugs to suppress MDR and increase chemosensitivity (Hashemi et al., 2021).

Recently, studies have confirmed that ginsenosides can be used in combination with a variety of drugs to enhance the inhibitory effect on cancer cells or increase the sensitivity to traditional chemotherapy drugs (Choi et al., 2013). For example, ginsenoside Rb1 and apatinib work synergistically to enhance the inhibition of growth of pharyngeal cancer cells (Li Y. et al., 2022). Combination therapy of ginsenoside Rg3 and chemotherapeutics drugs against BC has been demonstrated. Such as, ginsenoside Rg3 in combination with sorafenib has been shown to enhance the inhibitory effect on hepatocellular carcinoma growth by modulating HK2-mediated glycolysis and PI3K/Akt signaling pathways (Wei et al., 2022), whereas in combination with paclitaxel (PTX) and cisplatin (DDP) can enhance the anti-esophageal squamous cell carcinoma effect (Chang et al., 2014). Moreover, ginsenoside Rg3 also enhance the chemotherapy sensitivity of DDP-resistant human lung cancer cell and PTX-resistant TNBC by downregulating MDR-mediated proteins (including P-glycoprotein (P-gp), multidrug resistance-related protein (MPR1) and lung resistance protein 1 (LPR1)) (Liu et al., 2018) and inhibiting NF-κB signaling pathway, respectively (Yuan et al., 2017). Another ginsenoside Rh2, has been shown that in combination with regorafenib and more effectively inhibit the proliferation of liver cancer cells by regulating the expression of caspase-3 gene (Wang P. et al., 2020). In consistently, ginsenoside Rh2 can also inhibit the growth of human prostate cancer cells in combination with 1α, 25-dihydroxyvitamin D3 (Ben-Eltriki et al., 2021). In addition, lower doses of ginsenoside Rh2 combined with biotea protein A inhibited the proliferation, metastasis and invasion of BC cells by upregulating the expression of p53, p38 and ASK1, which was consistent with the effect of single drug administration (Ren et al., 2018). While *in vitro* and *in vivo* studies have shown that ginsenoside Rh2 can significantly enhance the anti BC effect of doxorubicin and reduce cardiotoxicity during the treatment phase (Hou et al., 2022).

Emerging studies demonstrate that multiple ginsenosides exhibit significant anti-cancer activity in several cancers *in vitro* and *in vivo*. In this review, we summarize the molecular mechanisms of various ginsenosides in inhibiting BC, including targeting miRNAs and their roles in inhibiting BC cells proliferation, inducing cell cycle arrest and apoptosis, inhibiting metastasis and invasion, and triggering autophagy aspects of the role. In addition, ginsenosides can also be used in combination with chemotherapeutic drugs to increase the chemosensitivity of drug-resistant cancer cells and enhance the anti-BC effect of chemotherapeutic drugs. In particular, a variety of ginsenosides can exert anti-BC effects by targeting miRNAs, among which ginsenoside Rh2 has been widely identified. From this, we speculate that ginsenosides have great potential as broad-spectrum anti-cancer drugs and effective chemosensitizers. However, with the continuous accumulation of evidence, more clinical studies are urgently needed for ginsenosides in the anti-BC mechanism and in improving oral bioavailability.

In summary, this review summarizes the molecular mechanism of ginsenosides including PPD, PPT and other three saponins inhibiting BC, among which the PPD type is the most reported. Thus, ginsenosides have great potential as broad-spectrum anti-BC drugs and effective chemosensitizers.
